# The Value of Contrast-Enhanced Magnetic Resonance Imaging Enhancement in the Differential Diagnosis of Hepatocellular Carcinoma and Combined Hepatocellular Cholangiocarinoma

**DOI:** 10.1155/2022/4691172

**Published:** 2022-09-15

**Authors:** Lun Lu, ChenCai Zhang, Xian Yu, Ling Zhang, YaYuan Feng, YuXian Wu, JinJu Xia, Xue Chen, RuiPing Zhang, Juan Zhang, Ningyang Jia, SiSi Zhang

**Affiliations:** ^1^Department of Radiology, Eastern Hepatobilliary Surgery Hospital, The Second Military Medical University, No. 225 Changhai Road Yangpu Area, Shanghai 200433, China; ^2^Department of Radiology, Nanfang Hospital, Southern Medical University, Guangzhou 510515, China; ^3^Key Laboratory for Biorheological Science and Technology of Ministry of Education (Chongqing University), Chongqing University Cancer Hospital, Chongqing 400044, China

## Abstract

**Background:**

The distinction between combined hepatocellular-cholangiocarcinoma (cHCC-CC) and hepatocellular carcinoma (HCC) before the operation has an important clinical significance for optimizing the treatment plan and predicting the prognosis of patients. Magnetic resonance imaging (MRI) has been widely used in the preoperative diagnosis and evaluation of primary liver malignant tumors.

**Purpose:**

The aim is to study the value of preoperative clinical data and enhanced MRI in the differential diagnosis of HCC and cHCC-CC and obtain independent risk factors for predicting cHCC-CC. *Study type*. Retrospective. *Population.* The clinical and imaging data of 157 HCC and 59 cHCC-CC patients confirmed by pathology were collected. *Field Strength/Sequence*. 1.5T; cross-sectional T1WI (gradient double echo sequence); cross-sectional T2WI (fast spin echo sequence, fat suppression); enhancement (3D LAVA technology). *Assessment*. The differences between the HCC and cHCC-CC patients were compared. *Statistic Tests*. Using the *t*-test, chi-square test, and logistic regression analysis, *P* < 0.05 was considered statistically significant.

**Result:**

1. CHCC-CC was more likely to show multiple lesions than HCC (28.81% vs. 10.83%, *P* = 0.001) and more prone to microvascular invasion (MVI) (36.31% vs. 61.02%, *P* < 0.001). However, HCC had a higher incidence of liver cirrhosis than cHCC-CC (50.85% vs. 72.61%, *P* = 0.003). 2. The incidence of nonsmooth margin was higher in the cHCC-CC group (84.75% vs. 52.23%, *P* < 0.001). The incidence of peritumor enhancement in the arterial phase was higher in the cHCC-CC group (11.46% vs. 62.71%, *P* < 0.001) 3. According to the multivariate analysis, arterial peritumor enhancement (OR = 8.833,95%CI:4.033,19.346, *P* < 0.001) was an independent risk factor for cHCC-CC (*P* < 0.001)). It had high sensitivity (62.71%) and specificity (88.54%) in the diagnosis of cHCC-CC. *Date Conclusions*. Liver cirrhosis and the imaging findings of GD-DTPA-enhanced MRI are helpful for the differential diagnosis of HCC and cHCC-CC. In addition, the imaging sign of peritumoral enhancement in the arterial phase has high sensitivity and specificity for the diagnosis of cHCC-CC.

## 1. Introduction

Combined hepatocellular-cholangiocarcinoma (cHCC-CC) is a rare primary liver malignant tumor, which was first reported by Allen and Lisa [1], accounting for about 0.4–14.2% of primary liver carcinomas (PLCs) [2, 3]. The histopathological diagnosis of cHCC-CC requires the presence of both hepatocellular carcinoma (HCC) and cholangiocarcinoma (CC), with transition zones of intermediate morphology. Some studies have shown that cHCC-CC has the biological behavior of both HCC and ICC and the prognosis is poor [4]. For many tumors that are prone to recurrence, an early and accurate diagnosis is of great significance for the selection of clinical treatment options [5, 6]. As an effective treatment for HCC [7], there is no consistent conclusion on the therapeutic effect of liver transplantation in cHCC-CC, though radical resection and liver transplantation are the main surgical treatments for HCC and cHCC-CC. Moreover, most studies believe that liver transplantation in patients with cHCC-CC does not improve the survival rate of these patients [8, 9].

However, if the HCC component of cHCC-CC and the CC component of cHCC-CC are treated with transarterial chemoembolization (TACE), percutaneous ethanol injections (PEI), and radiofrequency ablation (RFA) and other modalities before surgery, respectively, the HCC component of cHCC-CC and the CCA component of cHCC-CC can be treated by chemoembolization or transarterial radioembolization. Burden benefits cHCC-CC patients before liver resection or transplantation [10]. As a consequence, the distinction between cHCC-CC and HCC before the operation has an important clinical significance for optimizing the treatment plan and predicting the prognosis of patients.

Magnetic resonance imaging (MRI) has been widely used in the preoperative diagnosis and evaluation of primary liver malignant tumors [11–15]. The current study demonstrated the strong diagnostic performance of LI-RADS v2018 for predicting primary hepatic malignancy [16] .This study intends to evaluate the preoperative clinical data and GD-DTPA-enhanced MRI features (based on the morphological and enhanced features defined by LI-RADS) and to study their value in the differential diagnosis of HCC and cHCC-CC, as well as to find independent risk factors for predicting cHCC-CC, providing more useful information for the differential diagnosis of the two.

## 2. Materials and Methods

### 2.1. Patients

This study was a retrospective study, which was approved by our hospital with an exemption of informed consent. In addition, the enhanced MRI examination has been widely used in clinics to exempt patients from informed consent.

**Figure 1:**
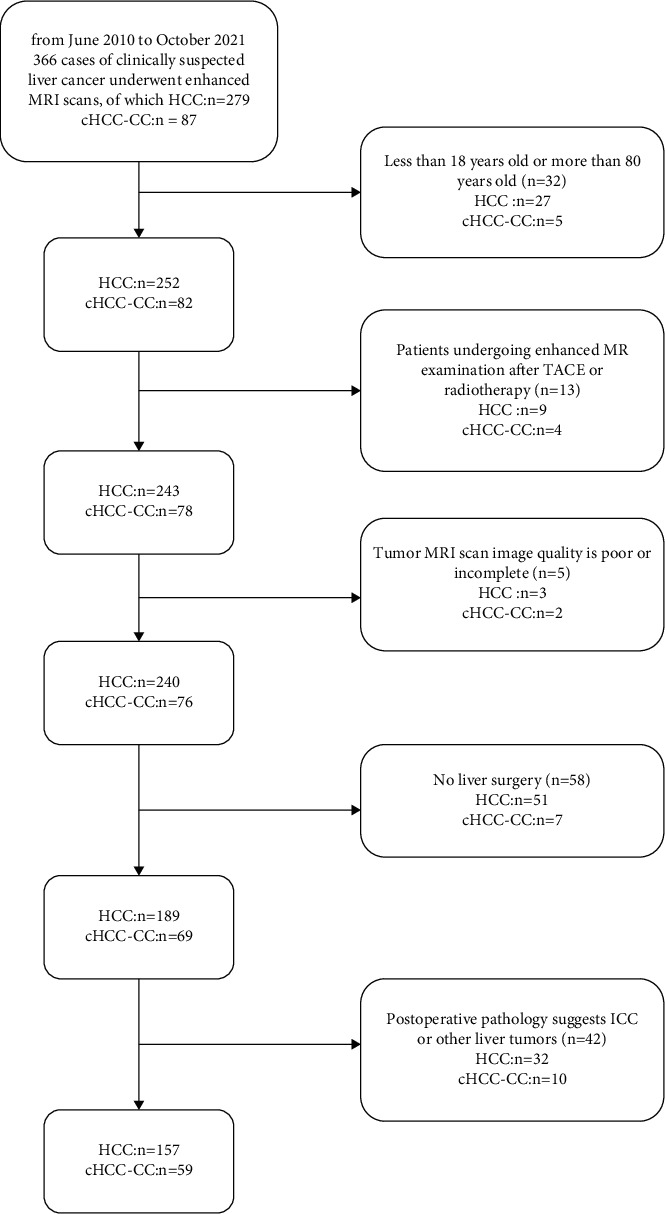
Flowchart detailing the patient selection process and exclusion criteria. In total, 157 patients with HCC and 59 patients with cHCC-CC were enrolled in the final analysis.

A retrospective collection of 366 cases of liver tumors from June 2010 to October 2021 in our hospital who underwent enhanced MRI scans was collected (HCC cases from January 2014 to October 2021 were selected for inclusion in the study), and 279 cases of HCC were obtained, 87 cases of cHCC-CC. The selection criteria were as follows: 1. The patients who aged 18–80 years old. 2. MRI suggested that a liver tumor of ≥1 cm was found. 3. Patients who underwent contrast-enhanced MRIs and were fully documented. 4. Surgical resection of the liver tumor was completed. 5. Postoperative pathology confirmed HCC or cHCC-CC. The exclusion criteria were as follows: 1. The patients whose age <18 years old or >80 years old. 2. The image quality of the tumor MRI scan was poor or the phase was not complete. 3. Those who did not undergo liver surgery. 4. Postoperative pathology revealed intrahepatic cholangiocarcinoma or other liver tumors. 5. Patients underwent contrast-enhanced MR examination after TACE or radiotherapy (Figure 1). After screening, 157 cases of HCC and 59 cases of cHCC-CC were finally included in the study.

### 2.2. MRI Examination and Evaluation

MR scanning parameters: GE optima MR360 was adopted with a field strength of 1.5T and an 8-channel abdominal surface coil. The patients fasted for 4 hours before scanning, and the patients were trained to breathe before scanning. The patients lay back on the inspection bed with their feet entering first. The 0.1 mmoL/Kg of gadolinium meglumine (GD-DTPA) was injected into the median cubital vein through a high-pressure injector with a flow rate of 2.0 ml/s. The enhanced scanning time was 22–28 s in the arterial phase, 50–70 s in the portal phase, and 90 –120 s in the delayed phase after injection of the contrast agent. Precontrast scan: cross-sectional T1WI: gradient double echo sequence was adopted, holding breath at the end of breath, with TR/TE = 190/(4.3,2.1) ms, slice thickness  = 6 mm, layer interval = 2 mm, matrix = 256 × 160, and FOV = 44 × 40 cm. DWI：*b* = 600 sec/mm^2^, cross-sectional T2WI: fast spin echo sequence, fat suppression, and respiratory gating were conducted, with TR/TE = 6667/85 ms, layer thickness = 6 mm, layer interval = 2 mm, matrix = 320 × 224, and FOV = 44 × 40 cm. The breath-holding scan was applied with TR/TE = 3000/74 ms, a layer thickness of 8 mm, the layer interval of 2 mm, matrix = 128 × 160, and FOV = 44 × 40 cm. Enhancement: cross-section adopted 3D LAVA technology, with TR/TE = 3.6/1.7 ms, layer thickness/layer spacing = 5/−2.5 mm, matrix: 256 × 192, and FOV = 44 × 40 cm.

Two radiologists engaged in abdominal imaging diagnosis for more than 10 years analyzed the MR appearance of all cases on the PACS system, including lesion size, boundary, capsule, smooth edge, arterial phase peritumoral enhancement, and so on. They would reach an agreement through discussion when in doubt.

The tumor size was measured by selecting the length and diameter of the largest plane according to the liver imaging reporting and data system 2018 standard [17], including the mass capsule, when the mass was shown most clearly in the MRI-enhanced portal phase images. Besides, the tumor edge was divided into smooth edges (nodular tumors with smooth edges) and nonsmooth edges (budding processes on cross-sectional and coronal images) [18]. The capsule was defined as the enhancement degree of the smooth edge of the tumor in the portal vein or delayed phase was higher than that of the mass, and pathologically, it was mainly fibrous components [19, 20]. The peritumoral enhancement in the arterial phase was defined as a crescent or polygonal enhancement area outside the edge of the tumor in the arterial phase. The degree of overall or partial enhancement was higher than that of the hepatic parenchyma. In addition, there was an extensive contact with the edge of the tumor, and the signal in the delayed phase was similar to that of the normal hepatic parenchyma [21]. Arterial rim enhancement is defined as a ring-shaped enhancement at the edge of the arterial mass.

### 2.3. Histopathological Analysis

The postoperative tumor tissues were standardized and stained with hematoxylin-eosin staining by an experienced pathologist (engaged in pathological diagnosis of liver cancer for 20 years). If necessary, immunohistochemical staining was performed. All pathological sections were reviewed according to the 2019 WHO classification, and the classification and diagnosis of HCC and cHCC-CC were performed [22]. That is, cHCC-CC is a mixed area of HCC and CC in tumor cells and there is a transitional area between the two types at the same time. The growth pattern of cancer focus included pericancerous infiltration, capsule formation, microvascular invasion (MVI), and satellite nodules, etc. Liver cirrhosis showed extensive fibrosis of liver tissues with pseudolobule formation.

### 2.4. Statistical Analysis

This study was divided into two groups with the following statistical methods:

Basic table: continuous variables (measurement data): the variables were consistent with normal distribution, using *t*-test and were presented as “mean + sd”; nonnormal distribution adopted a nonparametric test (Kruskal test) and was presented as “median (1/4–3/4IQR)”; classified variables (count/grade data) adopted the chi-square or Fisher test and were presented as “count (percentage)”.

Univariate and multivariate analysis: Logistic regression: SPSS19.0 statistical software package, and the chi-square test and the independent sample *t*-test were used for statistical analysis. At the same time, combined with clinical significance, the parameters were analyzed by a single multifactor logistic regression analysis, and the odds ratio (OR) and 95% confidence interval (CI) of each parameter were calculated. The results of multifactor analysis and statistically significant indicators were analyzed comprehensively. In addition, the sensitivity and specificity under different combinations were calculated. (*α* = 0.05, bilateral test).

## 3. Results

### 3.1. Patients

157 cases of HCC met the inclusion criteria, with an average age of 55.80 ± 10.44 and the male to female ratio was 134 : 23; 59 cases of cHCC-CC met the inclusion criteria, with a mean age of 52.80 ± 10.23, and the male to female ratio was 48 : 11. There was no significant difference in onset age and gender ratio between the two groups (*P*=0.067, 0.472). There was no significant difference in tumor diameter (defined by 5 cm), AFP, HBsAg, and HBeAg between HCC and cHCC-CC cases (*P*=0.116, 0.407, 0.589, and 0.159), respectively.

The proportion of multiple lesions in HCC is 10.83%. However, that of cHCC-CC was 28.81%. The probability of HCC complicated with liver cirrhosis was 72.61% and the probability of cHCC-CC was 50.85%. The differences between the two groups were statistically significant (*P*=0.001, 0.003 Table 1). At the same time, cHCC-CC is more prone to MVI than HCC manifestations(36.31% vs. 61.02%, *P* < 0.001Table 1).

### 3.2. Comparison of MRI Imaging Features

**Figure 2:**
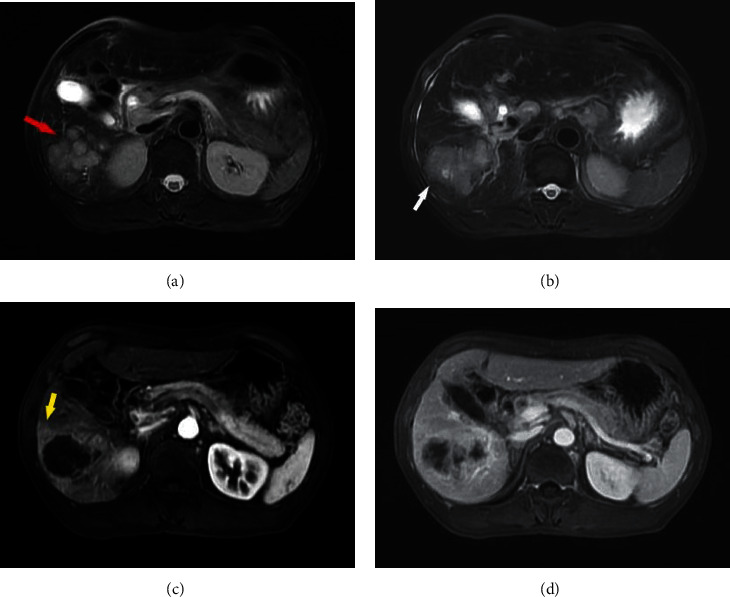
cHCC-CC presenting in a 42-year-old man. (a)-(b) multiple lesions (red arrow) and nonsmooth margin (white arrow) on T2WI; (c) the arterial phase shows obvious peritumoral enhancement (yellow arrow); and (d) without capsule shows in enhanced portal phase images.

**Figure 3:**
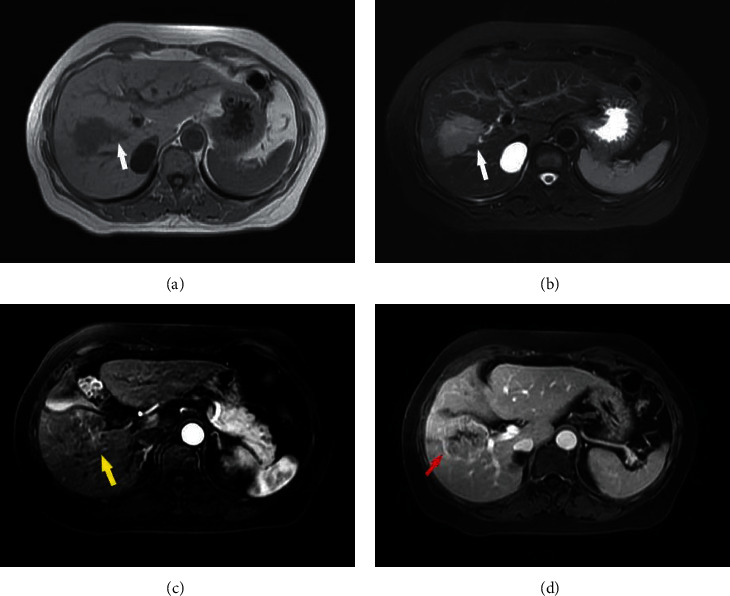
cHCC-CC presenting in a 51-year-old man. (a)-(b) nonsmooth tumor margin on T1WI, T2WI. (white arrow); (c) without obvious peritumoral enhancement in the arterial phase (yellow arrow); and (d) capsule obvious shows in enhanced portal phase images (red arrow).

**Figure 4:**
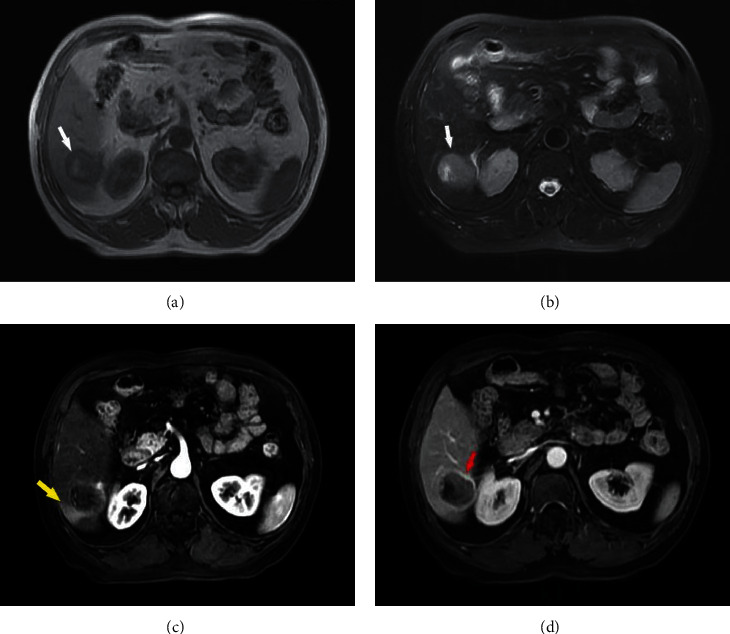
HCC presenting in a 58-year-old man. (a)-(b) smooth margin on T1WI, T2WI (white arrow); (c) the arterial phase shows obvious peritumoral enhancement (yellow arrow); and (d) capsule obvious shows in the enhanced portal phase images (red arrow).

There were significant differences in arterial peritumor enhancement and tumor margin between the HCC and cHCC-CC groups (*P* < 0.001). Besides, the incidence of nonsmooth margin in the cHCC-CC group was higher than that in the HCC group (84.75% vs. 52.23%, *P* < 0.001). Moreover, the HCC group had a lower incidence of peritumor enhancement in the arterial phase (11.46% vs. 62.71%, *P* < 0.001) The capsular appearance rates of the two groups on MRI were 40/157 (25.48%) and 8/59 (13.56%), respectively. There was no significant difference between the two groups (*P*=0.061, Table 1) (Figures 2–4).

### 3.3. Risk Factor Analysis

According to multivariate analysis, arterial peritumor enhancement (OR = 8.833,95%CI:4.033,19.346, *P* < 0.001) was an independent risk factor for cHCC-CC (*P* < 0.001) (Table 2). It had high sensitivity (62.71%) and specificity (88.54%) in the diagnosis of cHCC-CC (Table 3).

## 4. Discussion

cHCC-CC is a rare primary liver carcinoma. In general, the clinical and imaging manifestations of the disease are also different due to the difference in the volume of HCC cells and ICC cells in the tumor [23]. As a consequence, there is a high misdiagnosis rate in practical clinical work. This study mainly discusses the value of preoperative clinical data and MRI-enhanced imaging signs in the differential diagnosis of HCC and cHCC-CC.

It was found that there was no statistical significance in other aspects except the ratio of single focus to multiple lesions and the probability of mass complications with liver cirrhosis through the study of the clinical date of the two groups of patients. Lin et al. [24] found that the incidence of cHCC-CC in men is higher. However, in this study, these two types of liver cancer are not statistically significant in terms of gender and age. The author believes that it is related to the small number of cases collected. Clinical manifestations of HCC and cHCC-CC are not typical, and may only show skin itching, weight loss, and other symptoms [25]. Different patients have different sensitivity and attention to the changes in the body. As a consequence, patients come to see a doctor at different stages of the disease, so the difference between the two sizes is not statistically significant. Studies have shown that both HBsAg and HBeAg are closely related to the occurrence of hepatocellular carcinoma [26, 27]. AFP is mainly synthesized in the fetal liver, decreases gradually after birth and approaches the adult level in about a week, which is of great significance for the diagnosis of HCC [28]. However, it is usually related to the size of the tumor, especially when the tumor is smaller, the proportion of normal value can reach up to 35%–40% [29]. At the same time, some other lesions in the liver, such as hepatoblastoma, will also increase [30]. Because most of the cases collected in this study were smaller than 5 cm in diameter, and cHCC-CC contained both hepatocellular carcinoma and cholangiocarcinoma, there was no significant difference in AFP, HBsAg, and HBeAg between them. Most scholars believe that it originated from hepatic progenitor cells, though there are different opinions on the origin of cHCC-CC [2, 31]. Hepatic progenitor cells are a group of undifferentiated cells with hepatocyte function in the normal human liver, which are mainly distributed in the Hering duct of the portal vein and bile canaliculi [32]. On the other hand, the Hering duct constructs the relationship between the biliary system and the intralobular anatomy and microscopic system of the liver. Once cHCC-CC occurs, it is easy to have multiple lesions. At the same time, although the blood supply of cHCC-CC is less than that of HCC, its ability to invade large hepatic vessels, such as the portal vein and the hepatic vein, is similar to that of HCC. Besides, it is more likely to have a metastasis of peripheral lymph nodes [33]. As a consequence, the probability of multiple lesions in cHCC-CC is significantly higher than that in HCC, and the difference is statistically significant.

Cirrhosis, defined pathologically as multiple regenerative nodules surrounded by fibrous tissue, is the final evolutionary stage of all chronic progressive liver diseases [34], In the process of developing liver cirrhosis, due to the development of fibrous and regenerative tissues, many liver space-occupying lesions will change in shape and enhancement mode on the basis of the liver deformation, which brings difficulties in diagnosis [35]. Cirrhosis is the strongest risk factor for HCC [36].Studies have shown that up to 85%–90% of hepatocellular carcinoma patients have liver cirrhosis [37]. Although cHCC-CC contains liver cancer cells, liver cancer cells are only one of its components. As a consequence, the probability of cHCC-CC patients with liver cirrhosis is low and the difference between the two is statistically significant.

MRI-enhanced scanning is widely used in the diagnosis and differential diagnosis of benign and malignant liver lesions and the evaluation of liver function with the advantages of nonradiation and multimode imaging [38, 39]. It was found that there was a significant difference in tumor margin and peritumoral enhancement between the HCC and cHCC-CC groups. The analysis showed that the focus of HCC was mainly composed of hepatocellular carcinoma cells with few fibrous components. However, the fibrous tissue contained in cHCC-CC cholangiocarcinoma components would pull the mass in the process of growth and lead to lobular changes, so sometimes it could be shown as a budding protuberance on imaging [15]. Moreover, because cHCC-CC contains both HCC and ICC, HCC is more prone to hematogenous metastasis, and ICC is more likely to have lymph node metastasis. The invasiveness is stronger [33, 40], and the probability of a rough edge on the image is greater. The difference between the two is statistically significant. Studies have shown that [41, 42]. Peritumoral enhancement is an important index for predicting microvascular infiltration. It is generally believed that the main reason for this phenomenon is that the normal liver tissue is mainly supplied by the portal vein, and the tumor thrombus caused by the microvascular infiltration will lead to obstruction of the portal vein branches around the mass. As a result, the surrounding arteries are compensated [43]. As a consequence, when the enhanced scan is performed, the area can be obviously enhanced in the arterial phase. The analysis of the pathological results of the two groups showed that the microvascular infiltration ability of the cHCC-CC group was higher than that of the HCC group. Therefore, the cHCC-CC group was more likely to have a peritumoral enhancement in the arterial phase than the HCC group. The difference is statistically significant. The “Capsule sign” is one of the main signs of LI-RADS. It is mainly an imaging manifestation of collagen fibers produced by liver cancer cells, hepatocytes, and various factors activating stellate cells [19]. This sign has good heterogeneity in the diagnosis of HCC [44] and can better distinguish HCC from benign liver lesions or other non-HCC liver malignant lesions. The hepatoma cell components contained in cHCC-CC can also activate some stellate cells to produce collagen fibers, showing a “capsule sign” in the image. As a consequence, there is no significant difference between the two.

Through the multivariate analysis of the collected data, it was found that liver cirrhosis and arterial peritumor enhancement were independent risk factors for predicting cHCC-CC and the imaging feature of arterial peritumor enhancement had high sensitivity (62.71%) and specificity (88.54%) for the diagnosis of cHCC-CCA. It has certain hints for the diagnosis of cHCC-CC.

The main shortcomings of this study are as follows: 1. The number of cases was relatively small, which might have cause certain errors. 2. Because the imaging data provided by MRI plain scan was limited, the display of some lesions was poor, and the blood supply of lesions could not be well observed. As a consequence, this study did not choose the MRI plain scan data for analysis. 3. This study did not make a more detailed pathological classification of cHCC-CC. In the future, the author will conduct a more detailed study on these deficiencies.

## 5. Conclusions

Generally speaking, it can be concluded that by combining the two data, we can make a better differential diagnosis between HCC and cHCC-CC before operation according to the analysis of the preoperative clinical features and the imaging findings of the contrast-enhanced MRI. Compared with cHCC-CC, HCC is more likely to be complicated by liver cirrhosis. However, the incidence of multiple lesions with peritumoral enhancement in the arterial phase and rough margin is lower. Liver cirrhosis and arterial peritumoral enhancement are independent risk factors for predicting cHCC-CC. Arterial peritumoral enhancement has high sensitivity and specificity in the diagnosis of cHCC-CC.

## Figures and Tables

**Table 1:** Comparison of patient characteristics according to tumor type.

Characteristics	Total	HCC	cHCC-CC	*P*
*Sex*	216	157	59	
Male	182	134	48	0.472
Female	34	23	11	

Age	55.00 (10.44)	55.80 (10.44)	52.88 (10.23)	0.067

*Size(cm)*				0.116
<5	156	118	38	
≥5	60	39	21	

*AFP (ng/L)*				0.407
>0, ≤20	89	69	20	
>20, ≤400	79	55	24	
>400	48	33	15	

*HBsAg*				0.589
Negative	34	26	8	
Positive	182	131	51	

*HBeAg*				0.159
Negative	161	113	48	
Positive	55	44	11	

*Tumor number*				0.001
Single	182	140	42	
Multiple	34	17	17	

*Cirrhosis*				0.003
Negative	72	43	29	
Positive	144	114	30	

*MVI*				0.001
Negative	123	100	23	
Positive	93	57	36	

MRI features				
*Capsule*				0.061
Negative	168	117	51	
Positive	48	40	8	

*Arterial rim enhancement*				0.687
Negative	116	83	33	
Positive	100	74	26	

*Arterial peritumoral enhancement*				<0.001
Negative	161	139	22	
Positive	55	18	37	

*Tumor margin*				<0.001
Smooth	84	75	9	
Nonsmooth	132	82	50	

**Table 2:** Univariate and multivariate analyses of preoperative MR imaging findings in predicting the tumor type.

	Univariate analysis		Multivariate analysis	
	OR (95% CI)	*P*	OR (95%CI)	*P*
Tumor number	3.333 (1.566, 7.096)	0.002	2.393 (0.952, 6.012)	0.063
Cirrhosis	0.390 (0.210, 0.725)	0.003	0.373 (0.175, 0.793)	0.010
Capsule	0.459 (0.201, 1.049)	0.065	0.758 (0.278, 2.064)	0.587
Arterial rim enhancement	0.884 (0.484, 1.613)	0.687		
Arterial peritumoral enhancement	12.986 (6.316, 26.698)	<0.001	8.833 (4.033, 19.346)	<0.001
Tumor margin	5.081 (2.339, 11.037)	<0.001	2.356 (0.953, 5.824)	0.063

**Table 3:** Diagnostic performance of MR imaging findings in prediction of the tumor type.

	Sensitivity	Specificity	Accuracy	PPV	NPV
Cirrhosis	50.85% (30/59)	27.39% (43/157)	33.80% (73/216)	20.83% (30/144)	59.72% (43/72)
Arterial peritumoral enhancement	62.71% (37/59)	88.54% (139/157)	81.48% (73/216)	67.27% (37/55)	86.34% (139/161)
Combination of two findings (series connection)	28.81% (17/59)	90.45% (142/157)	73.61% (73/216)	53.13% (17/32)	77.17% (142/184)
Combination of two findings (parallel connection)	84.75% (50/59)	25.48% (40/157)	41.67% (73/216)	29.94% (50/167)	81.63% (40/49)

## Data Availability

The data used to support the findings of this study are available from the corresponding author upon request.
